# Gastrin stimulates a cholecystokinin-2-receptor-expressing cardia progenitor cell and promotes progression of Barrett's-like esophagus

**DOI:** 10.18632/oncotarget.10667

**Published:** 2016-07-18

**Authors:** Yoomi Lee, Aleksandra M. Urbanska, Yoku Hayakawa, Hongshan Wang, Andrew S. Au, Aesis M. Luna, Wenju Chang, Guangchun Jin, Govind Bhagat, Julian A. Abrams, Richard A. Friedman, Andrea Varro, Kenneth K. Wang, Malcolm Boyce, Anil K. Rustgi, Antonia R. Sepulveda, Michael Quante, Timothy C. Wang

**Affiliations:** ^1^ Division of Hematology and Oncology, Department of Medicine, Columbia University Medical Center, New York, NY, USA; ^2^ Division of Digestive and Liver Diseases, Department of Medicine, Columbia University Medical Center, New York, NY, USA; ^3^ Department of General Surgery, Zhongshan Hospital, Fudan University, Shanghai, China; ^4^ Department of Pathology and Cell Biology, Columbia University Medical Center, New York, NY, USA; ^5^ Biomedical Informatics Shared Resource, Herbert Irving Comprehensive Cancer Center, New York, NY, USA; ^6^ Department of Biomedical Informatics, Columbia University Medical Center, New York, NY, USA; ^7^ Department of Physiology, Institute of Translational Medicine, University of Liverpool, Liverpool, England; ^8^ Division of Gastroenterology and Hepatology, Mayo Clinic, Rochester, MN, USA; ^9^ Hammersmith Medicines Research, Central Middlesex Hospital, London, UK; ^10^ Division of Gastroenterology, Departments of Medicine and Genetics, Abramson Cancer Center, Perelman School of Medicine, University of Pennsylvania, Philadelphia, PA, USA; ^11^ Medical Clinic II, Clinic of the Right Bank, Technical University of Munich, Munich, Germany

**Keywords:** Barrett's esophagus, esophageal cancer, gastrin, gastrin receptors, stem cells

## Abstract

**Objective:**

The incidence of esophageal adenocarcinoma (EAC) is increasing, but factors contributing to malignant progression of its precursor lesion, Barrett's esophagus (BE), have not been defined. Hypergastrinemia caused by long-term use of proton pump inhibitors (PPIs), has been suggested as one possible risk factor. The gastrin receptor, CCK2R, is expressed in the cardia and upregulated in BE, suggesting the involvement of the gastrin-CCK2R pathway in progression. In the *L2-IL-1β* mouse model, Barrett's-like esophagus arises from the gastric cardia. Therefore, we aimed to analyze the effect of hypergastrinemia on CCK2R+ progenitor cells in *L2-IL-1β* mice.

**Design:**

*L2-IL-1β* mice were mated with hypergastrinemic (*INS-GAS*) mice or treated with PPIs to examine the effect of hypergastrinemia in BE progression. *CCK2R-CreERT* crossed with *L2-IL-1β* mice were used to analyze the lineage progenitor potential of CCK2R+ cells. Cardia glands were cultured *in vitro*, and the effect of gastrin treatment analyzed. *L2-IL-1β* mice were treated with a CCK2R antagonist YF476 as a potential chemopreventive drug.

**Results:**

Hypergastrinemia resulted in increased proliferation and expansion of Barrett's-like esophagus. Lineage tracing experiments revealed that CCK2R+ cells are long-lived progenitors that can give rise to such lesions under chronic inflammation. Gastrin stimulated organoid growth in cardia culture, while CCK2R inhibition prevented Barrett's-like esophagus and dysplasia.

**Conclusions:**

Our data suggest a progression model for BE to EAC in which CCK2R+ progenitor cells, stimulated by hypergastrinemia, proliferate to give rise to metaplasia and dysplasia. Hypergastrinemia can result from PPI use, and the effects of hypergastrinemia in human BE should be studied further.

## INTRODUCTION

The incidence of esophageal adenocarcinoma (EAC) has risen 6-fold in the U.S. since the 1970s [1, 2]. Major risk factors for EAC include male gender, obesity, and a history of gastroesophageal reflux disease (GERD), but Barrett's esophagus (BE) increases the risk of EAC by 11-fold [3–5]. In BE, present in up to 1.6% of unselected populations, the normal squamous epithelium of the lower esophagus is replaced by columnar epithelium. However, the risk of EAC in patients with nondysplastic BE is quite low (0.12% annually) [6]. Screening for dysplasia in BE patients has not been shown to decrease mortality [7, 8]. Thus, a better understanding is needed of the factors involved in neoplastic progression of BE.

Proton pump inhibitors (PPIs) have been used routinely over the last several decades to treat BE patients, but their impact on progression of BE remains unresolved. PPIs ameliorate reflux symptoms and promote healing of esophagitis in most patients over the short term [9], and most studies suggest they reduce BE progression. However, PPIs cause both acid suppression and a physiologic secondary hypergastrinemia, which can lead to extremely high plasma gastrin levels in some patients [10, 11]. In one study that stratified PPI-treated BE patients by gastrin levels, the highest quartile of gastrin levels was associated with an increased risk of high-grade dysplasia or EAC on biopsy [12]. However, the effect of hypergastrinemia and overactive downstream signaling in BE and EAC has not yet been demonstrated in experimental models.

The effects of gastrin are mediated by the CCK2R (also known as CCKBR), a G-protein coupled receptor expressed in the central nervous system and throughout the gastrointestinal tract [13]. In the gastric corpus of the proximal stomach, the receptor mediates acid secretion as well as growth and differentiation of the epithelium. [13–15] CCK2R is upregulated in the mucosa during ulcer healing, and inhibition impairs mucosal regeneration. [16, 17] While CCK2R might in theory mark a short-lived progenitor cell that expands during healing, we have recently shown that CCK2R marks a +4 gastric antrum progenitor, which is Lgr5^neg or low^ and lineage traces entire antral glands [18]. These CCK2R+ progenitors in the gastric antrum can undergo interconversion to Lgr5+ cells and contribute to gastric cancer development. However, the role of CCK2R in the gastric cardia and BE has not been clarified.

CCK2R is upregulated 2-fold in human BE tissues, and mediates gastrin-induced proliferation and anti-apoptotic effects in primary cultures of BE biopsies [19]. In addition, CCK2R is upregulated in the gastric cardia of mice with Barrett's metaplasia, and this effect is enhanced with administration of bile acids [20]. Studies in animal models of gastrointestinal cancer have demonstrated that CCK2R signaling can accelerate tumorigenesis *in vivo*, such as in gastrin-overexpressing *INS-GAS* mice that develop proximal gastric cancers [21].

The *L2-IL-1β* transgenic mouse model develops Barrett's-like metaplasia in response to constitutive expression of the pro-inflammatory cytokine IL-1β in the squamous mucosa of the esophagus and forestomach [20]. Mouse and human anatomy differ in that squamous mucosa in mouse stomach extends partway into the anatomical stomach; however metaplasia and dysplasia develop at the gastroesophageal junction in humans and the *L2-IL-1β* mice develop a similar phenotype at the squamocolumnar junction, with changes throughout the junction between the forestomach/esophagus and the columnar stomach. In this model, gastric progenitors migrate from below the squamocolumnar junction (SCJ) proximally into the esophagus, giving rise to columnar and mucus (similar to intestinal) metaplasia. Inducible lineage tracing has demonstrated that Barrett's-like lesions in *L2-IL-1β* mice can arise from Lgr5+ cells in the cardia, although other progenitor markers including CCK2R, were also upregulated in the cardia [20]. Thus, while cardia glands may be structurally similar to antral glands and express CCK2R, the precise role or expression pattern of CCK2R in the cardia and Barrett's esophagus has not been defined.

## RESULTS

### Hypergastrinemia accelerates the development of Barrett's-like metaplasia in mice

**Figure 1:**
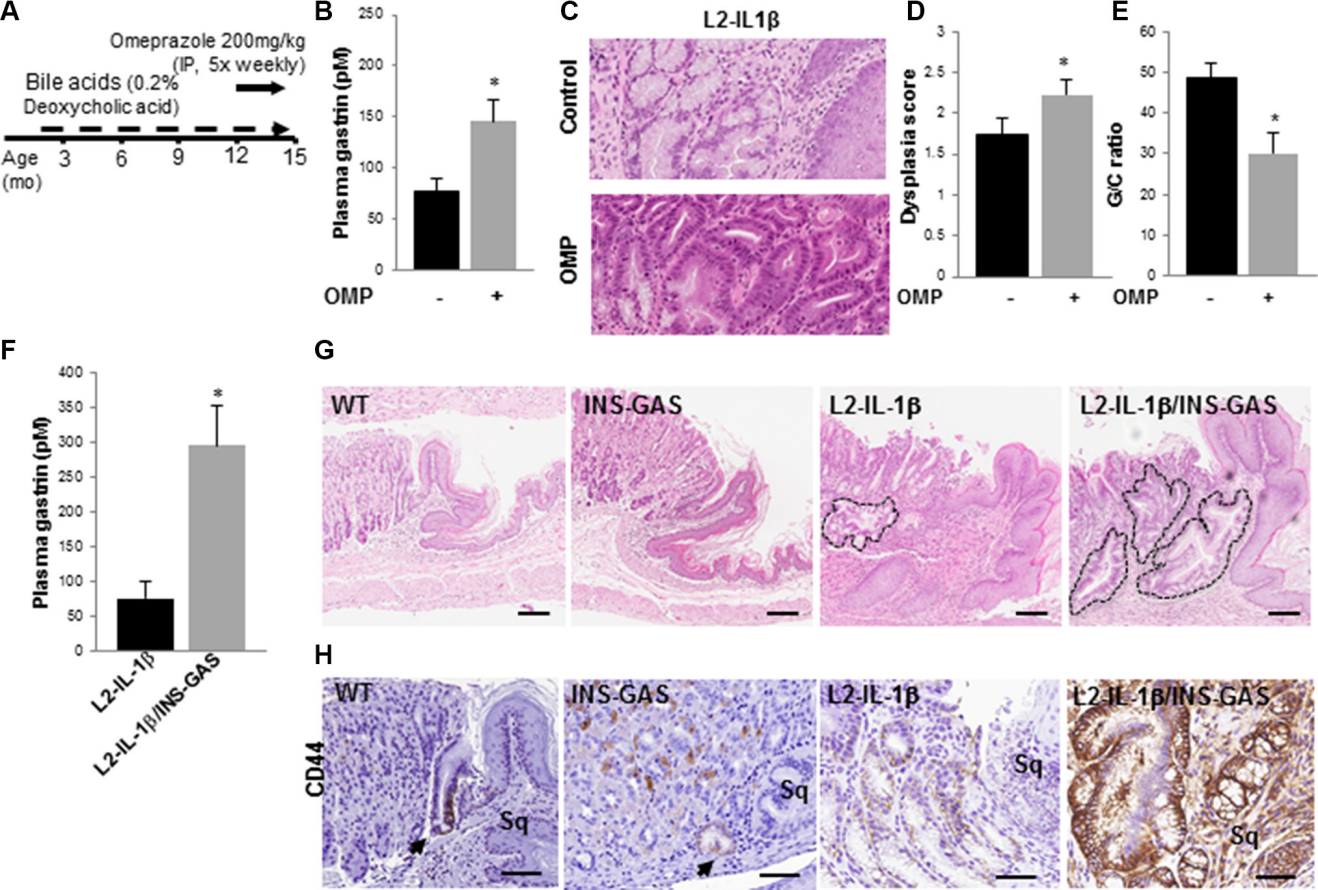
Hypergastrinemia accelerates Barrett's-like esophagus in *L2-IL-1β* mice (**A**) Schematic for omeprazole (OMP) treatment in wild type and *L2-IL-1β* mice. Mice were started on deoxycholic acid (0.2%) in their drinking water at 8 weeks of age, and then given omeprazole 200 mg/kg intraperitoneally 5 days a week, from 12 – 15 months of age. (**B**) Gastrin levels (pM) in *L2-IL-1β* mice (*N* = 5) and *L2-IL-1β* mice treated with omeprazole (*N* = 5) (*p* = 0.037).(**C**) Representative H&E stained sections from BE-like lesions at the SCJ of control and omeprazole-treated *L2-IL-1β* mice. (**D**) Dysplasia scores of BE-like lesions in control (*N* = 5) and OMP-treated (*N* = 5) *L2-IL-1β* mice (*p* < 0.05). (**E**) Goblet-like cell to columnar cell (GC) ratio from BE-like lesions in control (*N* = 5) and OMP-treated (*N* = 5) *L2-IL-1β* mice (*p* < 0.05). (**F**) Plasma gastrin levels (pM) in *L2-IL-1β* mice (*N* = 3) and *L2-IL-1β/INS-GAS* mice (*N* = 6) (*p* = 0.03). (**G**) H&E stained histological sections from the SCJ from wild type, *INS-GAS*, *L2-IL-1β*, *L2-IL-1β/INS-GAS* mice at 12 months of age. Outlined areas indicate BE mucinous metaplasia at the SCJ. Scale bars = 100 um. (**H**) CD44 immunostaining of cardia cells in WT, *INS-GAS*, *L2-IL-1β*, *L2-IL-1β/INS-GAS* mice. Scale bars = 100 um.

In order to test the effect of PPI treatment and subsequent hypergastrinemia on development and progression of Barrett's-like esophagus, bile acid-treated WT and *L2-IL-1β* mice were given omeprazole for three months (Figure 1A). Omeprazole treatment increased plasma gastrin levels compared to untreated controls, as seen in human patients (Figure 1B). As reported previously, bile acid treatment induced hyperplastic mucosa extending proximal to the SCJ. Omeprazole treatment accelerated metaplastic and dysplastic changes, leading to increased mitotic figures, loss of cell polarity, and hyperchromatic nuclei (Figure 1C). Omeprazole-treated mice had higher dysplasia scores and a reduced percentage of mucus producing cells compared to untreated mice (Figure 1C–1E). Barrett's-like esophagus with increased dysplasia has lower levels of mucus cell differentiation, with fewer cells resembling the goblet cells in human BE, and thus the G/C ratio (goblet-like cell/columnar cell) quantifies this lesser degree of cellular differentiation.

While PPIs strongly suppress acid secretion, leading to numerous changes in gastric physiology, we hypothesized that PPI-related hypergastrinemia was primarily responsible for the acceleration of BE in mice. To address the role of genetically-induced hypergastrinemia on the progression of BE, we crossed *INS-GAS* mice, overexpressing amidated gastrin under the transcriptional control of the rat insulin promoter [22], with *L2-IL-1β* mice. Plasma gastrin levels were significantly higher in *L2-IL-1β/INS-GAS* mice compared to *L2-IL-1β* mice (Figure 1F). Hypergastrinemia alone was not sufficient to induce metaplastic or dysplastic cells at the SCJ of *INS-GAS* mice, whereas the addition of hypergastrinemia to the chronic inflammation/BE phenotype led to a marked expansion of Barrett's-like esophagus in *L2-IL-1β/INS-GAS* mice compared to *L2-IL-1β* mice (Figure 1G). This expanded BE occurred in a region where we previously did not observe metaplasia or dysplasia in *INS-GAS* mice [22]. Mucus cell differentiation was reduced in *L2-IL-1β/INS-GAS* mice compared to *L2-IL-1β* mice, suggesting that hypergastrinemia inhibits the intestinal differentiation typically seen in BE patients. We have previously shown that inhibition of Notch signaling through a gamma-secretase inhibitor (GSI) can inhibit proliferation and induce more classical intestinal differentiation in the *L2-IL-1β* mouse model [20]; this was also true for *IL-1β/INS-GAS* mice, where GSI-treatment led to a marked increase in Muc-2 and Alcian blue-positive metaplastic cells in the mouse cardia (Figure S2), consistent with an intestinally differentiated Barrett's-like phenotype. CD44, a potential progenitor cell marker, is normally expressed in undifferentiated cells near the base of cardia glands, and is also found in gastric dysplasia and cancer. In our model, CD44+ cells were expanded in *L2-IL-1β* mice, with a further increase in CD44+ cells in *L2-IL-1β/INS-GAS* mice (Figure 1H), supporting the notion that hypergastrinemia promotes the development of a less differentiated, hyperproliferative type of Barrett's-like esophagus, with a low G/C ratio.

### Gastrin-dependent expansion of CCK2R+ cells in Barrett's-like metaplasia

**Figure 2:**
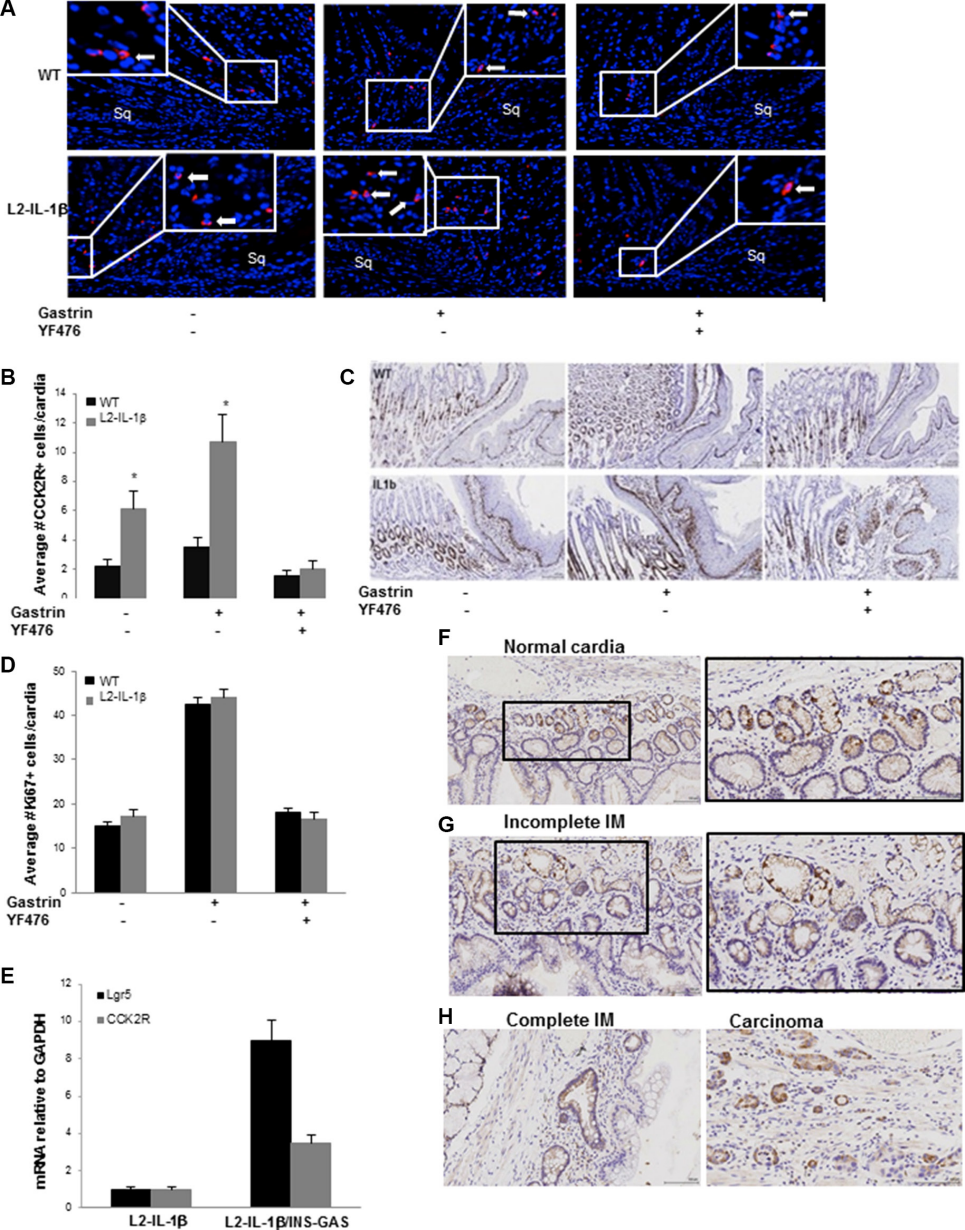
Gastrin-dependent expansion of CCK2R+ cells in Barrett's-like metaplasia (**A**) Immunofluorescence for CCK2R (red) at the SJC in WT and *L2-IL-1β* mice treated with or without gastrin infusion, YF476 or both. See Supplementary Methods for details of treatment. Sq indicates esophageal squamous mucosa at the SCJ. Arrows indicate CCK2R+ cells. (**B**) Quantification of CCK2R+ cells per cardia gland in WT and *L2-IL-1β* mice treated with or without gastrin infusion, YF476 or both. (*N* = 5) (**C**) Representative sections showing Ki67 immunostaining from WT and *L2-IL-1β* mice treated with gastrin infusion with or without YF476. (**D**) Quantification of Ki67+ cells/cardia gland in WT and *L2-IL-1β* mice treated with or without gastrin infusion, YF476 or both. (*N* = 3) (*p* = 0.01). (**E**) RT-PCR for Lgr5 and CCK2R in the cardia of *L2-IL-1β/INS-GAS* mice (*N* = 6) compared to *L2-IL-1β* controls (*N* = 5) (*p* = 0.007 -CCK2R, *p* = 0.03 -Lgr5). (**F**) CCK2R immunostaining of normal human gastric cardia. (**G**) CCK2R immunostaining of human BE tissue with incomplete intestinal metaplasia (IM). (**H**) CCK2R immunostaining of human BE tissue with complete IM and adenocarcinoma.

Since Barrett's-like esophagus appeared to be accelerated by hypergastrinemia, we examined the expression of the gastrin receptor (CCK2R) in epithelial cells in the mouse cardia and in human BE. First we examined CCK2R expression in wild type mouse gastric cardia by immunohistochemistry, which showed rare cells in the cardia glands (Figure 2A). Examination of the *L2-IL-1β* mouse cardia showed some expansion of CCK2R+ cells in the inflamed cardia (Figure 2A, 2B). To further test the effect of gastrin in these CCK2R+ cells, WT and *L2-IL-1β* mice were infused with gastrin (5 ug/kg/h/day) and/or a CCK2R antagonist YF476 for 7 days. While gastrin treatment had a minimal effect on the number of CCK2R+ cells in WT mice, the number of CCK2R+ cells in *L2-IL-1β* mice was increased by gastrin infusion (Figure 2A, 2B). Epithelial proliferation in the gastric cardia as measured by Ki67 immunostaining was increased by gastrin infusion, and blocked by simultaneous YF476 administration (Figure 2C, 2D). Interestingly, the expression of cardia stem cell marker *Lgr5* was markedly upregulated in hypergastrinemic mouse cardia, along with *CCK2R* (Figure 2E).

We next stained various human biopsy samples for *CCK2R* by *in situ* hybridization. Similar to the mouse stomach, the normal human cardia contains CCK2R+ cells near the base of the glands (Figure 2F). The numbers of CCK2R+ cells in human BE, relative to the gastric cardia, are increased with either incomplete and complete intestinal metaplasia, and further evident in esophageal adenocarcinoma arising from BE (Figure 2G–2H). These results suggest that CCK2R+ cells can expand in cardia glands during BE progression in response to hypergastrinemia.

### CCK2R marks long-lived progenitors in cardia and gives rise to Barrett's-like metaplasia

**Figure 3:**
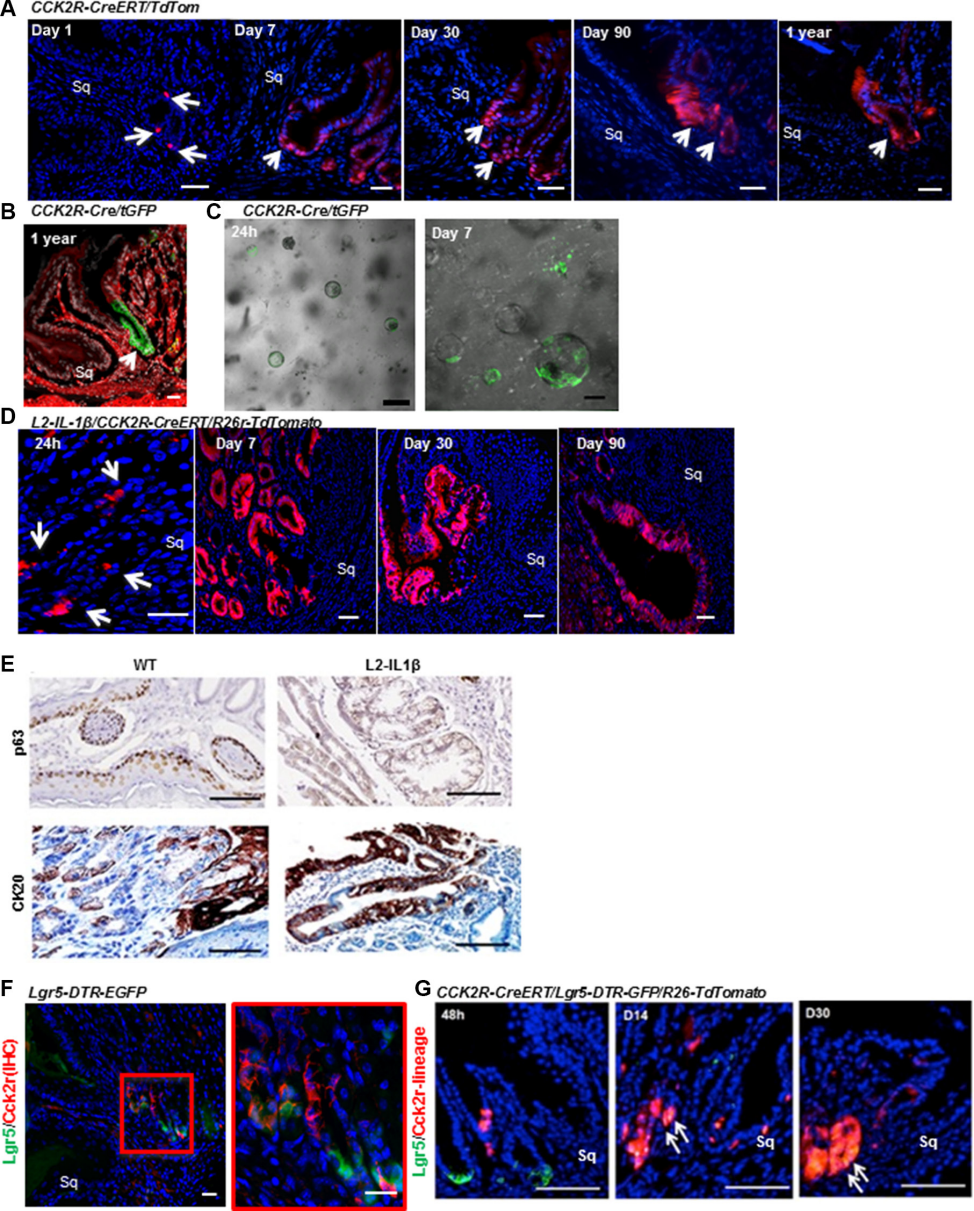
CCK2R marks long-lived progenitor cells in the cardia that give rise to Barrett's-like metaplasia (**A**) *CCK2R-CreERT/TdTom* mice were tamoxifen-induced at 6 weeks of age, then sacrificed after 1, 7, 30, 90 and 360 days. Sq indicates squamous epithelium. Scale bars= 100 um. (**B**) CCK2R marks the cardia in *CCK2R-Cre/tGFP* mice. Green: recombined CCK2R+ cells; red: non-recombined. Scale bars = 100 um. (**C**) Organoids grown for 24 h (left) and 7 days (right), isolated from the cardia of tamoxifen-treated *CCK2R-CreERT/tGFP* mice. Scale bars = 100 um. (**D**) *L2-IL-1β/CCK2R-CreERT/R26r-TdTomato* mice were tamoxifen-induced at 6 weeks of age, then sacrificed after 1, 30, 90, and 360 days. Blue: DAPI. Red: CCK2R-CreERT recombined cells. Sq indicates squamous epithelium. Scale bars = 100 um. (**E**) Immunohistochemical staining for p63 (top) and CK20 (bottom) in 1-year old WT and *L2-IL-1β* mice. Scale bars = 100 um. (**F**) Immunofluorescence for CCK2R in *Lgr5-DTR-EGFP* mouse cardia showing rare cells expressing both Lgr5 (green) and CCK2R (red). Red box is magnified on the right. Blue: DAPI. Sq indicates squamous epithelium. Scale bars = 100 um. (**G**) Immunofluorescence images from *CCK2R-CreERT/Lgr5-DTR-GFP/R26-TdTomato* mice induced with tamoxifen and analyzed at 2, 14 and 30 days. Arrows show double positive (Lgr5+ and CCK2R traced) cells. Scale bars = 100 um.

We then sought to understand the role of CCK2R+ cells in the cardia of wild type mice and in Barrett-s like esophagus in *L2-IL-1β* mice. We hypothesized that CCK2R+ cells might act as progenitors in the cardia (as previously demonstrated in the gastric antrum [23]) and analyzed the cell fate of CCK2R+ cells in mice. We utilized *CCK2R-CreERT; R26-TdTomato* mice [23] and examined lineage tracing of CCK2R+ cells by administering tamoxifen at 6 weeks of age. Similar to CCK2R staining, rare recombined cells were visible near the base of the cardia 24 hours after induction (Figure 3A). By days 7 and 30, these Tomato-red cells expanded and moved upward in the cardia gland until almost all the cells in the gland were labeled. Tomato-red cells persisted for up to one year, showing that CCK2R+ cells are long-lived gastric progenitors in cardia (Figure 3A). Similar tracing events were observed when another *Rosa26* reporter line *CCK2R-CreERT; Rosa26rmTmG* mice were used (Figure S3). Another mouse line expressing constitutive Cre-recombinase under the CCK2R promoter also confirmed the expression in cardia progenitors (Figure 3B). We isolated and cultured cardia glands from *CCK2R-CreERT/R26-mTmG* mice after tamoxifen induction and observed that CCK2R+ cells rapidly expanded and lineage traced cultured cardia organoids (Figure 3C), indicating that these cells represent progenitor cells with lineage tracing capability.

To determine whether Barrett's-like esophagus arises from CCK2R+cells in the cardia, we crossed *L2-IL-1β* to *CCK2R-CreERT/R26-TdTomato* mice. In these mice, *TdTomato* expression was present in cardia 24 hours after tamoxifen induction, and within a few weeks expanded to entire BE glands. In bile-acid treated *L2-IL-1β/CCK2R-CreERT/R26-TdTomato* mice, CCK2R+ cells expanded towards the squamocolumnar junction within 90 days (Figure 3D). At one year, the lineage-traced lesions in the cardia of *L2-IL-1β* mice were CK20-positive and p63-negative metaplasia (Figure 3E). These results suggest that CCK2R+ progenitors from the cardia can give rise to Barrett-s like esophagus in response to chronic inflammation.

In previous studies we showed that *LGR5* is upregulated in biopsies of human BE, and *Lgr5*+ stem cells can lineage trace the cardia and BE lesions in *L2-IL-1β* mice. Since both *Lgr5* and CCK2R mark cardia progenitor cells, we tested whether there is any overlap between *Lgr5*+ and CCK2R+ cells in the cardia gland. We stained *Lgr5-DTR-GFP* mouse stomach for CCK2R, and confirmed that the majority of CCK2R+ cells in the cardia (approximately 85%) are *Lgr5*-negative (Figure 3F). Thus, these represent two distinct stem cell pools that can lineage trace the cardia gland. We next generated *CCK2R-CreERT/Lgr5-DTR-GFP/R26-TdTomato* mice, and administered tamoxifen. At early time points, there were no recombined red cells in the *Lgr5*+ green population; however, CCK2R+ stem cells gave rise to Lgr5+ cells at the base after 30 days (Figure 3G). A similar expression pattern for *Lgr5* and CCK2R lineages was demonstrated by *in situ* hybridization in *CCK2R-CreERT/R26-TdTomato* mice (Figure S4), suggesting the presence of interconversion between these two initially distinct populations.

### Amidated gastrin promotes cardia organoid growth through CCK2R

**Figure 4:**
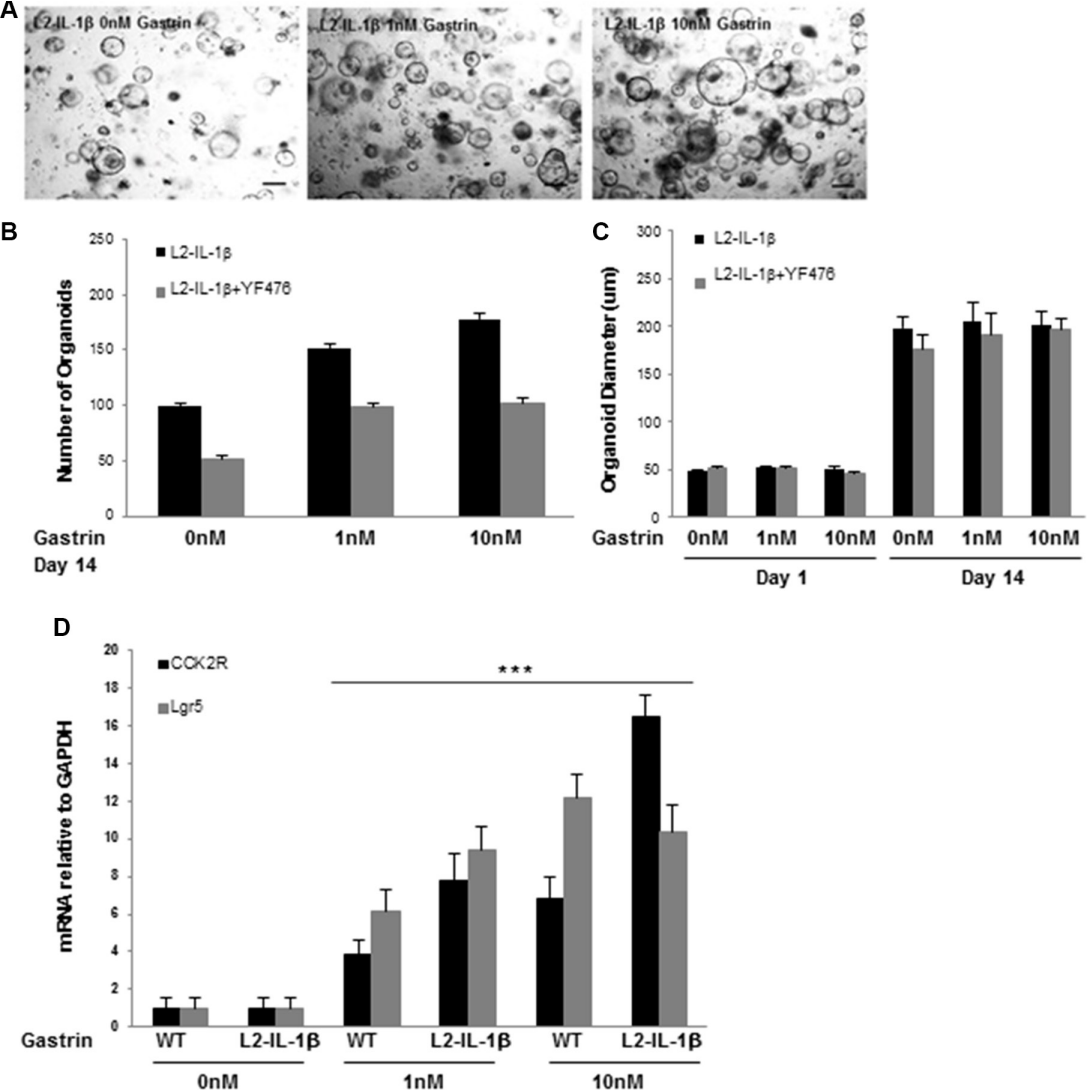
Gastrin promotes cardia organoid growth through CCK2R (**A**) Organoids grown from the gastric cardia of *L2-IL-1β* mice in 0 nM, 1 nM, 10 nM gastrin for 14 days. Scale bars =100 um. (**B**) Quantification of organoid number after 14 days of growth in 0 nM, 1 nM, or 10 nM gastrin (*p = 0.01 0 nM vs 10 nM gastrin) from the gastric cardia *L2-IL-1β* mice. Treatment with YF476 (12 mg/mL) blocked the effect of gastrin on organoid number (*p* < 0.05 for all three gastrin doses). (**C**) Quantification of organoid diameter at day 1 and day 14 of growth in 0 nM, 1 nM, or 10 nM gastrin, in *L2-IL-1β* mice, with or without YF476 (12 mg/mL). YF476 had no effect on organoid diameter. (**D**) RT-PCR for CCK2R or Lgr5 from organoids of WT or *L2-IL-1β* cardia grown for 14 days in 0 nM, 1 nM or 10 nM gastrin. (***p < 0.001 for all 4 dataset points: 1 nM gastrin WT and *L2-IL-1*β when compared to 0 nM gastrin WT and *L2-IL-1*β for CCK2R and Lgr5, respectively; and for 10 nM gastrin WT and *L2-IL-1*β when compared to 0 nM gastrin WT and *L2-IL-1*β for CCK2R and Lgr5, respectively).

In order to confirm that gastrin can promote progression of Barrett's-like esophagus in a direct manner by inducing proliferation in CCK2R+ cells, we studied cardia organoids from *L2-IL-1β* and WT mice. Organoids grown from the *L2-IL-1β* mouse cardia increased in number in response to gastrin in a dose-dependent manner (Figure 4A–4B). There was no increase in organoid size with gastrin treatment (Figure 4C). The effect of gastrin on the number of *L2-IL-1β* cardia organoids was blocked by YF476, highlighting the specificity of gastrin/CCK2R signaling on organoid expansion. The expression of *Cck2r* and *Lgr5* was significantly increased in gastrin-treated organoids in a dose-dependent manner compared to untreated organoids (Figure 4D), suggesting that amidated gastrin activates and expands the cardia progenitor compartment.

### Blockade of CCK2R prevents the development of Barrett's-like metaplasia in mice

**Figure 5:**
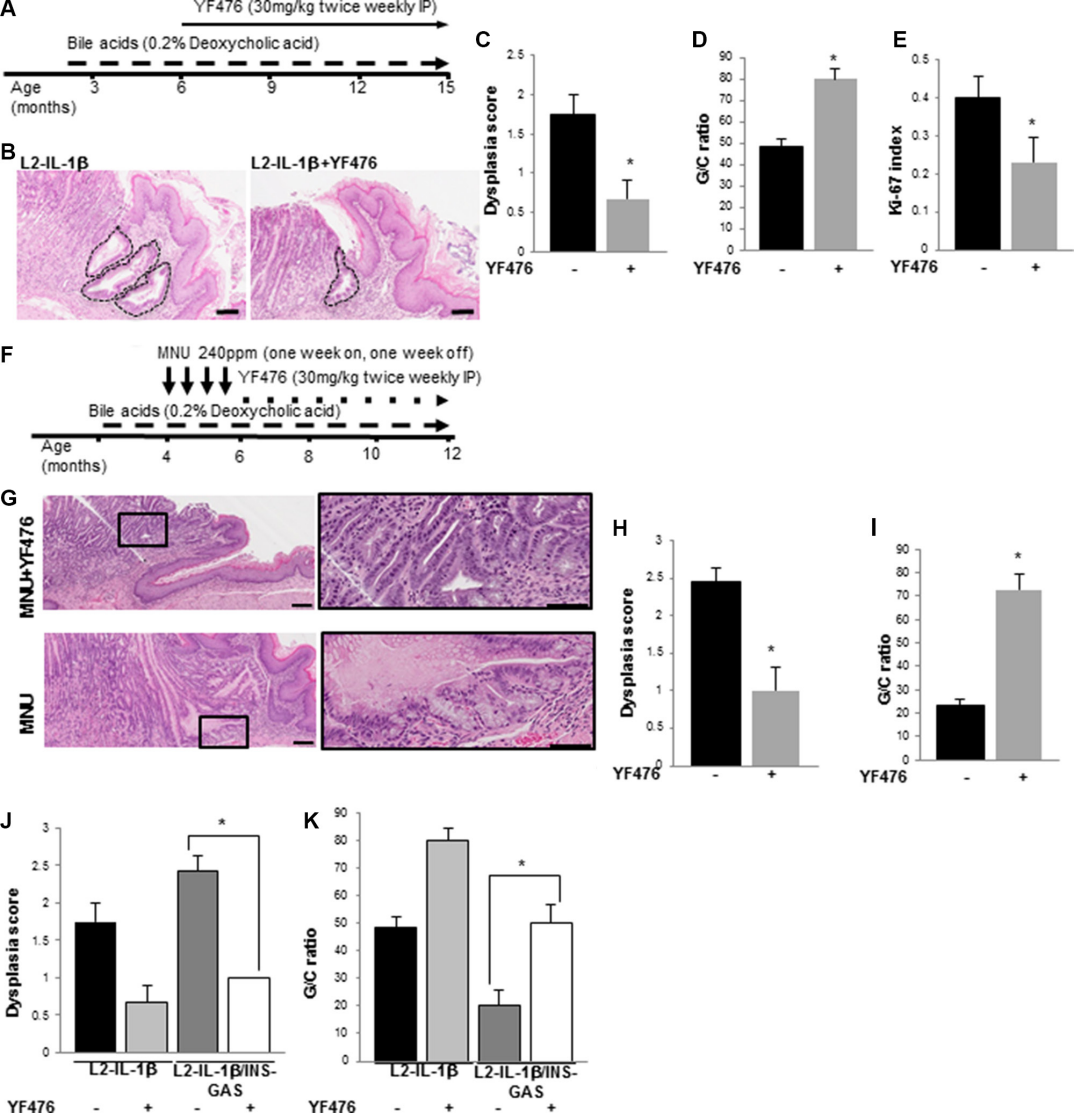
CCK2R blockade prevents the development of Barrett's-like esophagus in *L2-IL-1β* mice (**A**) Treatment schematic for *L2-IL-1β* mice with bile acid and YF476. Mice were started on deoxycholic acid (0.2%) in drinking water starting at 8 weeks of age, and then given YF476 (30 mg/kg) intraperitoneally twice a week starting at 6 months of age. (**B**) Representative H&E stained histopathology sections showing extent of BE involvement in YF476-treated and untreated *L2-IL-1β* mice. Scale bars = 100 um. (**C**) Dysplasia scores in YF476-treated (*N* = 7) and untreated (*N* = 7) *L2-IL-1β* mice (*p* = 0.03). (**D**) Goblet cell (GC) ratio in YF476-treated (*N* = 7) and untreated (*N* = 7) *L2-IL-1β* mice (*p* = 0.03). (**E**) Ki-67 index in the cardia of YF476-treated (*N* = 7) and untreated (*N* = 7) *L2-IL-1β* mice (*p* = 0.01). (**F**) Treatment schematic for *L2-IL-1β* mice with bile acid, MNU, and YF476. Mice were started on deoxycholic acid (0.2%) in drinking water starting at 8 weeks of age, and then given MNU (240 ppm) in the drinking water starting at 4 months of age with one week on and one week off for 4 cycles. Thereafter, mice were treated with YF476 (30 mg/kg) by IP twice a week. (**G**) Representative H&E stained histopathologic sections showing extent of BE involvement in MNU-treated *L2-IL-1β* mice with (top) or without (bottom) YF476. Boxed area on left is magnified on right. Scale bars = 100 um. (**H**) Dysplasia scores in MNU-treated *L2-IL-1β* mice with (*N* = 7) or without (*N* = 8) YF476. (*p* = 0.03). (**I**) G/C ratio in MNU-treated *L2-IL-1β* mice with (*N* = 7) or without (*N* = 8) YF476 (*p* = 0.03). (**J**) Dysplasia scores in *L2-IL-1β* (*N* = 8), *L2-IL-1β* with YF476 (*N* = 7), *L2-IL-1β/INS-GAS* (*N* = 7), *L2-IL-1β/INS-GAS* with YF476 (*N* = 7) (*p < 0.05). (**K**) G/C ratios in *L2-IL-1β* (*N* = 8), *L2-IL-1β* with YF476 (*N* = 7), *L2-IL-1β/INS-GAS* (*N* = 7), *L2-IL-1β/INS-GAS* with YF476 (*N* = 7) (*p < 0.05).

To investigate whether gastrin blockade prevents the progression and proliferation of Barrett's-like esophagus cells, we treated *L2-IL-1β* mice with YF476 (Figure 5A) at 6 months of age, when the first Barrett's-like changes are observed at the SCJ. Mice were sacrificed at 15 months of age. CCK2R blockade increased systemic levels of gastrin in a compensatory manner (not shown). Nevertheless, YF476 treatment led to a decrease in the dysplasia score, increased differentiated goblet-like cell to columnar cell (G/C) ratio, and decreased Ki67 index in cardia (Figure 5B–5E).

*L2-IL-1β* mice treated with bile acids and MNU develop dysplastic lesions at an earlier age than mice treated with bile acids alone, and thus MNU/bile acid-treated *L2-IL-1β* mice were also treated with YF476 or vehicle (Figure 5F). YF476 prevented the development of advanced dysplasia and markedly increased the mucus cell (G/C) ratio compared to controls (Figure 5G–5I). Furthermore, when *L2-IL-1β/INS-GAS* mice were treated with YF476, the dysplasia score in *L2-IL-1β/INS-GAS* mice was decreased and the mucus cell (G/C) ratio increased, indicating a strict dependence on CCK2R activation in these mouse phenotypes (Figure 5J, 5K).

## DISCUSSION

We demonstrate here that PPI-induced or genetic hypergastrinemia can increase the Barrett's-like metaplasia phenotype in our *L2-IL-1β* mouse model, and that this can lead to accelerated progression to dysplasia. Blocking CCK2R with a small molecule inhibitor (YF476) in *L2-IL-1β* mice inhibited this effect. We have further shown that CCK2R, which is upregulated in human BE tissue, marks a long-lived gastric cardia progenitor cell that expands in the setting of chronic inflammation and hypergastrinemia and gives rise to Barrett's-like esophagus in the *L2-IL-1β* mouse model. Hypergastrinemia could stimulate CCK2R+ cells in BE tissue to proliferate, and increased proliferation is correlated with less differentiation, less mucus (goblet-like) cell metaplasia in BE areas, and with accelerated malignant transformation. These findings suggest that elevated serum gastrin levels in BE patients warrants further study.

Importantly, hypergastrinemia enhanced the development of dysplasia within Barrett's-like esophagus, particularly in MNU-treated animals. These findings are consistent with some human data, where BE patients with the highest gastrin levels were more likely to have a history of high-grade dysplasia or EAC [12]. It is possible that indirect effects of gastrin or PPIs could have had this effect. Hypergastrinemia is also known to induce acid secretion, which by itself could in theory have caused an accelerated phenotype in the *INS-GAS* mice. However, mice do not develop reflux esophagitis, and we have in the past excluded such a mechanism, since acidified water in the *L2-IL-1β* mouse model did not increase metaplasia and dysplasia [20]. Moreover, the fact that PPI treatment produced a similar phenotype to gastrin overexpression (in *INS-GAS/L2-IL-1β* mice) does point to the likelihood of direct effects of gastrin. Our data raises the possibility that hypergastrinemic states may worsen BE in patients with preexisting esophageal inflammation through providing a more carcinogenic niche that enables increased proliferation and less differentiation.

Most epidemiologic studies suggest that PPIs reduce the risk of progression from BE to HGD or EAC, although there are no randomized placebo-controlled clinical trials demonstrating that PPIs have chemopreventive effects. The effect of PPIs on G/C ratio in BE patients has not been analyzed to our knowledge. A recent meta-analysis reported that PPI use in BE was associated with a significant reduction in the risk of progression to HGD or EAC (OR 0.29) [24]. However, 95–98% of all identified BE patients are treated with PPIs, and as such prior studies have lacked well-matched control groups. [24–26] Additionally, there has been some inconsistency with regard to the effects of PPI use on progression. In a nested case-control study using a Veterans' Affairs database, PPI use was not associated with a reduced risk of EAC after adjusting for NSAID and statin use and other potential confounders [26]. Case-control studies from the UK and from Denmark have reported an increased risk of EAC in BE patients on PPIs [27, 28]. Importantly, few of the studies to date have accounted for gastrin levels in assessing the effects of PPIs on the risk of progression in BE. In a cross-sectional study of BE patients on PPIs by Wang *et al*, those with the highest gastrin levels were significantly more likely to have a history of HGD or EAC [12]. If hypergastrinemia can contribute to carcinogenesis in BE, this has implications for continuing PPIs in patients who develop high gastrin levels on treatment.

However, PPIs have significant benefits in human BE, and omeprazole might worsen Barrett's-like esophagus in mice more so than in humans. Unlike humans, mice do not have acid reflux, which may promote proliferation and dysplasia [29], and which is reduced by PPIs [30, 31]. In humans, omeprazole decreases acid secretion and reduces duodenogastric reflux [32]. In *L2-IL-1β* mice, however, the inflammatory esophagitis is genetically induced and maintained not by GERD but by the constitutive expression of IL-1β in the esophagus, rendering the esophagitis refractory to PPI treatment. Though this is a limitation of our model, *L2-IL-1β* mice were treated with bile acid in the drinking water to promote esophagitis, mimicking a mechanism by which BE can be accelerated by PPIs, as hypergastrinemia induces reduced gastric motility and therefore potentially more bile acid reflux.

Hypergastrinemia in *L2-IL-1β/INS-GAS* mice led to increased stem cell markers in the cardia. We [20] and others [33] have previously demonstrated that *Lgr5* marks a long-lived stem cell in the cardia that can become activated in response to chronic inflammation. We show here that CCK2R also marks a long-lived progenitor cell in the cardia, similar to our earlier finding that CCK2R marks progenitors in the gastric antrum [18]. Cardia organoids from *L2-IL-1β* mice proliferate in response to gastrin stimulation, suggesting a model in which CCK2R+ cells are “primed” by adjacent inflammation to proliferate in response to gastrin. In addition to marking cardia progenitor cells, CCK2R+ cells also gave rise to Barrett's-like metaplasia and dysplasia in *L2-IL-1β* mice. Thus, CCK2R represents a second marker linking Barrett's-like metaplasia to its origin in the gastric cardia. CCK2R is not expressed at appreciable levels in the small intestine, yet numerous studies have now documented high levels of expression in human BE [19, 34]. similar to the increased cardia expression in our *L2-IL-1β* mice. Indeed, the finding that CCK2R+ cells can give rise to Barrett's-like esophagus in mice is consistent with recent reports that human Barrett's glands have an organizational structure similar to gastric glands, and probably start out within gastric metaplasia prior to acquiring intestinal features [35].

The transition from a normal CCK2R+ progenitor to a Barrett's-like progenitor appeared to be largely driven by chronic inflammation. In our model, expression of IL-1β provided the necessary stimulus for SCJ inflammation that led to upregulation of CCK2R. While we could not verify whether IL-1β directly or indirectly induced CCK2R expression in cardia cells, our data strongly suggest that inflammation in the cardia due to the presence of IL-1β increased the number of gastrin-responsive progenitor cells. *L2-IL-1β* mice had a greater number of organoid-forming progenitors in the gastric cardia compared to WT mice, consistent with an expansion of potential progenitors that could give rise to metaplasia and dysplasia.

The expression of the CCK2R in the cardia appears to have functional significance. CCK2R expression is increased in the gastric mucosa in response to cryoinjury and other forms of gastric ulceration, where it contributes to the regenerative response to injury [16]. Epithelial cell proliferation during ulcer healing is inhibited by CCK2R blockade and stimulated by omeprazole and exogenous gastrin; however, CCK2R expression in this setting is typically transient and quickly downregulated [17]. Gastrin stimulation appears to have the same proliferative effect on Barrett's epithelium, but in our model system this phenomenon was more long lasting.

In conclusion, our data suggest that hypergastrinemia promotes of progression and dysplasia in Barrett's-like esophagus in our mouse model. The limitations of this mouse model warrants caution in extrapolating these data to human BE. However these findings suggest that in patients with BE, high gastrin levels warrant further longitudinal study and consideration of either trials of CCK2R inhibitors or more selective use of PPIs.

## MATERIALS AND METHODS

### Mice

*L2-IL-1β* mice were generated as described previously in a C57BL/6 background [20]. Eight-week old mice were given drinking water containing bile acids (0.2% deoxycholic acid, pH 7.0, Sigma, St. Louis, MO) for the duration of the study. Omeprazole (Sigma, St. Louis, MO) 200mg/kg/day was given intraperitoneally 5 times weekly in a 1:1 mixture of dimethyl sulfoxide (DMSO) and PEG 300 at a concentration of 40mg/mL. *INS-GAS*, *CCK2R-CreERT*, *CCK2R-Cre*, *Lgr5-DTR-eGFP, Rosa26rLacZ*, *Rosa26rTdTom*, and *Rosa26rmTmG* mice have been previously described [23]. Tissues from cardia were isolated as described in Figure S1. All murine experiments were performed in compliance with federal laws and institutional guidelines and approved by the IACUC of the Columbia University Animal Care Facility (protocol AAAF-2657).

In lineage tracing studies, tamoxifen 5 mg was given by oral gavage. The gastrin receptor blocker, YF476 (kindly provided by Dr. Keiji Miyata and Dr. Hidenobu Yuki, Yamanouchi Pharmaceutical Co. Ltd., Tsukuba, Japan) was dissolved in PEG-300 (Sigma, St. Louis, MO) at 12 mg/mL (30 mg/kg) were given intraperitoneally twice weekly.

N-methyl-nitrosourea (MNU, Sigma, St. Louis, MO) was administered at 240 ppm in the drinking water for one week, followed by non-medicated water for one week, repeated 5 times over a total of 10 weeks. After 10 weeks, drinking water with bile acids was resumed.

## SUPPLEMENTARY MATERIALS



## Abbreviations

BE: Barrett's Esophagus
CCK2R: Cholecystokinin 2 receptor
EAC: Esophageal Adenocarcinoma
GERD: Gastroesophageal reflux disease
IL1β: interleukin-1β
MNU: N-methyl-nitrosourea
PCR: polymerase chain reaction
PPI: Proton Pump Inhibitors
SCJ: Squamocolumnar Junction
WT: wild type
