# Supplementary material for: Gastrin stimulates a cholecystokinin-2-receptor-expressing cardia progenitor cell and promotes progression of Barrett's-like esophagus
Source: Oncotarget. 2016 Jul 18;8(1):203–14. doi: 10.18632/oncotarget.10667 (PMC5352112; doi:10.18632/oncotarget.10667)
Supplement: Supplementary file 1 [file oncotarget-08-203-s001.pdf]

Gastrin stimulates a cholecystokinin-2-receptor-expressing cardia progenitor cell and promotes progression of Barrett's-like esophagus

Supplementary Materials

METHODS

Mice

All mouse studies and breeding were carried out under the approval of IACUC of Columbia University. Animals were free of specific murine pathogens, and housed in microisolator, solid-bottomed polycarbonate cages, fed a commercially prepared pelleted diet, and given water *ad libitum*.

Wild type, *L2-IL-1β* and *L2-IL-1β/CCK2R-CreERT* mice underwent a surgery during which osmotic pumps (Alzet, Cupertino, CA) were implanted under dorsal skin filled with gastrin or saline (control). The dose of gastrin was adjusted to 5 ug/kg/h for 7 days (Sigma). Upon completion of gastrin infusion, mice were sacrificed and their tissues collected. Three month-old wild type (WT) and *L2-IL-1β /INS-GAS* mice were injected intraperitoneally with gamma-secretase inhibitors (DBZ) for 7 days at a dose of 5 umol/kg (Tocris Bioscience, Bristol, UK). DBZ was suspended finely in 0.5% (w/v) hydroxypropylmethylcellulose (Methocel E4M) and 0.1% (w/v) Tween 80 in water. Mice were sacrificed and tissues collected and fixed for immunostaining.

Quantitative polymerase chain reaction

At the time mice were sacrificed, portions of forestomachs and esophagi (above the squamocolumnar junction) and portions of stomach (below the squamocolumnar junction) were frozen in Trizol and kept at −80°C. RNA was isolated and reverse-transcribed using the SuperScript III First-Strand Synthesis system. Quantitative PCR was performed using QuantiTect SYBR Green PCR dye (Qiagen, Valencia, CA) and the ABI General System 7300 (ABI Applied System, University Park, IL). The PCR conditions were: 95°C for 3 minutes, then cycles of 95°C for 30 seconds, 55°C for 30 seconds, and 72°C for 30 seconds for a total of 40 cycles.

Histopathologic analysis

The stomachs and esophagi from mice were fixed overnight in 10% formalin or 4% paraformaldehyde overnight followed by 30% sucrose, embedded in paraffin or OCT compound, and processed by standard histological methods. They were cut into 4um sections as illustrated in Figure S1, and stained with hematoxylin and eosin (H&E). Histopathologic scoring was performed by Dr. Govind Bhagat of the Department of Pathology and Cell Biology at Columbia University Medical Center. Criteria for scoring included a microscopic dysplasia score (0 = no dysplasia, 1 = superficial epithelial atypia, 2 = atypia in glandular complexity, 3 = low grade dysplasia, 4 = high grade dysplasia) and a macroscopic evaluation which was performed at the time of sacrifice (0 = no abnormalities at SCJ, 1 = focal < 10% tumors or nodularity along SCJ, 2 = partial 10–50%, 3 = >50%, 4 = continuous tumors along SCJ). The extent of metaplasia was examined along the entire SCJ, although lesions were most numerous at the GEJ. The gastric cardia was defined as the first 2–3 proximal gastric glands adjacent to the squamous epithelium of the esophagus or forestomach which do not contain parietal or chief cells. Histopathologic score was also independently assessed in a blinded manner by Michael Quante of the Technical University of Munich.

Calculation of the goblet-like cell/columnar cell ratio (G/C ratio)

Mouse tissue was stained with PAS and the area of mucus producing cells and non-mucus producing columnar cells was evaluated in the overall metaplastic region of the mouse Barrett metaplasia at the SCJ. The ratio of mucus producing (goblet-like) and columnar cells was calculated by the following method: every lumen surrounded by columnar-lined cells was assumed to represent one Barrett crypt. Each crypt was scored as (+) or (−) for mucus producing goblet-like cells. The mucus to columnar or GC-ratio was then defined as the number of positive (+) Barrett crypts divided by the total number of Barrett crypts. Data from the mean of 3 SCJ sections per mouse was calculated.

Immunohistochemistry/Immunofluorescence

Immunohistochemical studies were performed with avidin-biotin-peroxidase kits (Vector Laboratories, Burlingame, CA). We used the following primary antibodies: Ki67, CCK2R (AbCam), CD44 (BD Biosciences), p63 (Santa Cruz Biotechnology), Muc-2 (Thermo Scientific), and CK20 (Developmental Studies

Hybridoma Bank (DSHB). The primary and secondary antibodies were used at 1/100 dilution unless otherwise stated. Primary antibodies were incubated at 4°C overnight, in a humidified chamber. Subsequently, the sections were incubated with biotinylated secondary antibodies (Vectastain ABC kit; Vector Laboratories, Burlingame, CA) for 30 minutes, followed by incubation with avidin-coupled peroxidase (Vector Laboratories) for 30 minutes. Diaminobenzidine (DAB; Dako) as the chromogen and slides were counterstained with Mayer's hematoxylin. For immunofluorescence, Alexa fluor 488 or 555 secondary antibodies (Invitrogen) were used and then counterstained with 4′, 6-diamidino-2-phenylindole (Vector Laboratories).

RNAscope *in situ* hybridization (ISH)

RNAscope analysis for Lgr5 mRNA was performed on formalin fixed paraffin fixed sections using the Advanced Cell Diagnostics Inc. (ACD) kit according to the manufacturer's instructions with the following modification, the HybEZ™ Slide Rack was placed in the HybEZ™ Humidity Control Tray, covered with lid and inserted into the humidified chamber for 2 HRS at room temperature.

Morphometric analysis

Size of organoids were measured and quantified with free software Fiji (ImageJ, NIH, Bethesda, MD).

Cardia organoid culture

Stomachs of 8-week old mice were removed, opened longitudinally along the greater curvature, washed in cold PBS, and the gastric cardia was dissected out using a razor blade. For the schematic of the dissected tissue, please refer to Figure S1. Tissues were minced into 0.5mm pieces and added to a buffer composed of Dulbecco's modified Eagle medium (DMEM) with 2.5% FBS, penicillin/streptomycin (Invitrogen), 1 u/mL collagenase type V (Sigma, St. Louis, MO), 125 ug/mL dispase type II (Invitrogen) for 1 hour digestion. Afterwards, samples were centrifuged at 900 rpm for 5 minutes at 4°C and supernatant removed. Cells were resuspended and washed with PBS+10% FBS four times, then passed through a 70-um cell filter (BD Biosciences). Cells were embedded in Matrigel on ice at 5000 cells/25 uL Matrigel per well (BD Biosciences) and seeded in 48-well plates. Matrigel was polymerized for 10 minutes at 37°C, and 250 uL/well basal culture medium added. Culture medium consisted of advanced DMEM /F12 medium containing penicillin/streptomycin, 10 mM HEPES, Glutamax, N2, B27 (all from Invitrogen) and 1mM N-acetylcysteine (Sigma), EGF 50 ng/mL (Invitrogen), Noggin 100 ng/mL (Peprotech), R-spondin1 500ng/mL (R&D), Wnt3a 100 ng/mL (Peprotech). Human Gastrin I (Sigma) was added in the concentrations described. Media was changed every 48 hours. After 14 days organoids were imaged and the number counted and the diameters measured.

Microscopy

All specimens were acquired using an Eclipse TU2000-U microscope (Nikon) connected to a cooled color CCD camera (Diagnostic Instruments) using SPOT software (Nikon Inc., Melville, NY). *In vitro* organoid culture was imaged using 2-photon microscopy (Nikon, A1P).

Human tissue samples

Human tissue was obtained from the pathology department at Columbia University, which included gastric and esophageal tissue from endoscopic biopsies, endoscopic mucosal resections and surgical resections. Histologic review and assessment was performed by a gastrointestinal pathologist (A.R.S.) and included normal cardia, Barrett's with incomplete intestinal metaplasia, Barrett's with complete intestinal metaplasia, and esophageal adenocarcinoma. Four specimens with each diagnosis were stained with H&E to confirm the diagnosis, and then stained with antibodies to the CCK2R, and representative images obtained.

Plasma gastrin

The gastrin concentration in plasma was measured by (A.V.) through radioimmunoassay [36].

Statistical analysis

The differences between means were compared using Student's *t*-test or Mann-Whitney *u*-test. *P* values < 0.05 were considered to indicate statistical significance. Error bars denote the mean ± SEM (standard error mean).

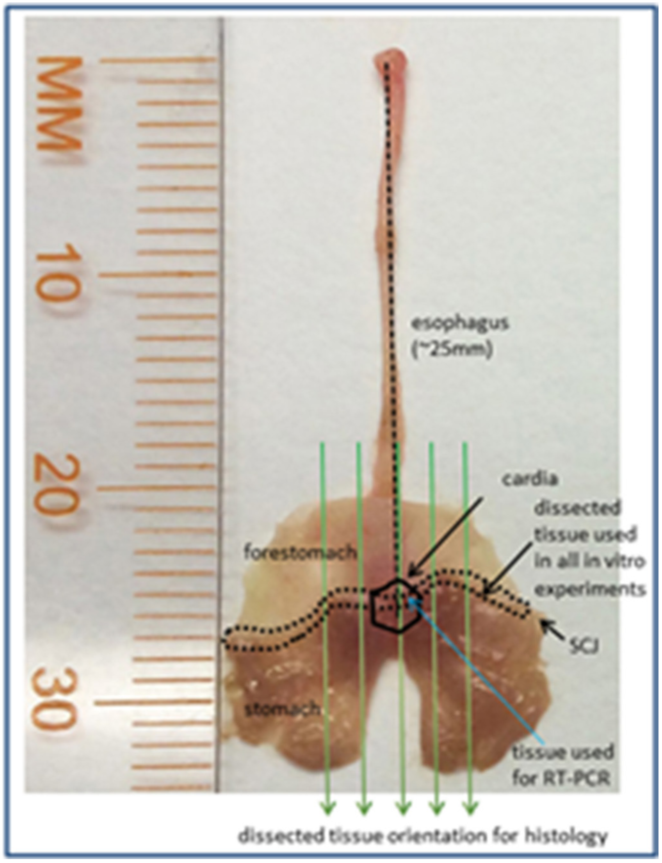

Supplementary Figure S1: Anatomy and dissection of the murine GE junction. Schematic figure explaining the location of dissected tissue used for *in vitro* organoid and RT-PCR experiments.

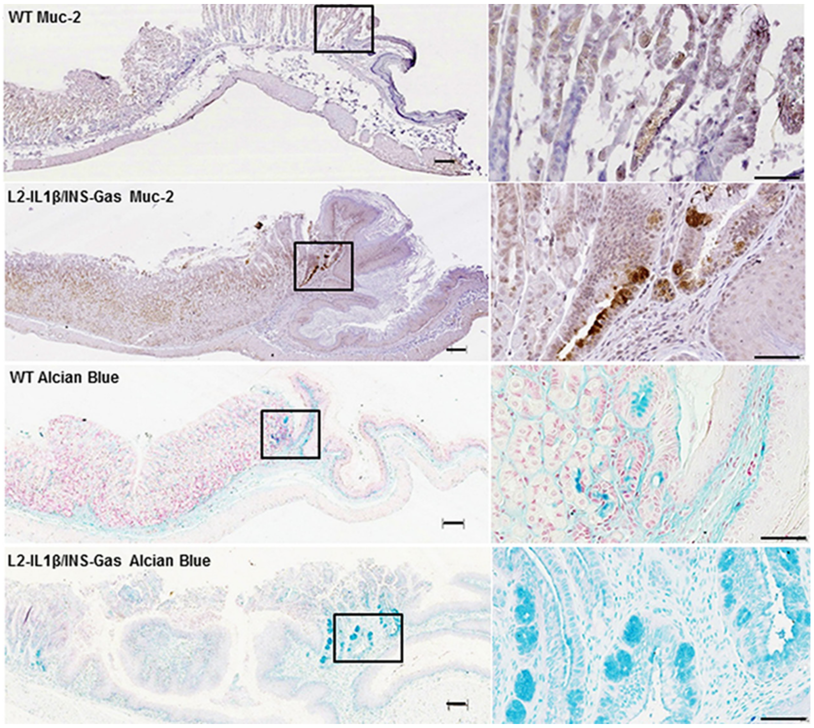

Supplementary Figure S2: Notch inhibition results in intestinal-type differentiation in murine Barrett's. Muc-2 (top) and Alcian blue (bottom) staining in wild type (WT) and *L2-IL-1β /INS-GAS* mice treated with γ-secretase inhibitors (DBZ) at a dose of 5 umol/kg by i.p. injection for 7 days. Scale bars indicate 100 um.

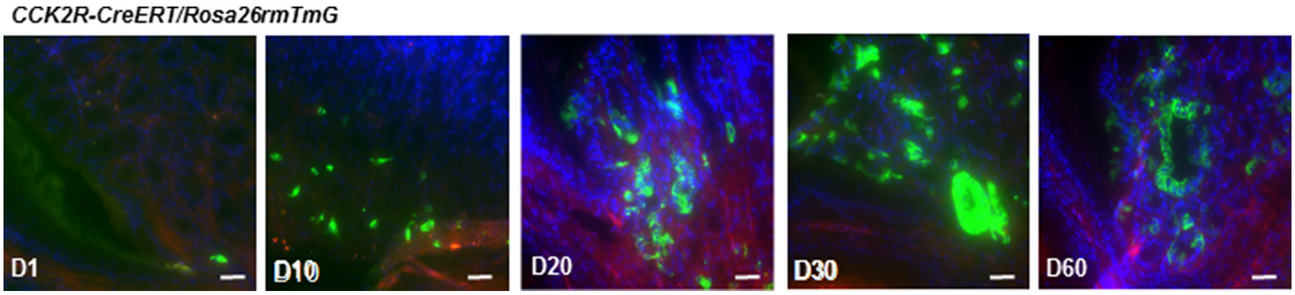

Supplementary Figure S3: CCK2R lineage traces the gastric cardia. *CCK2R-CreERT/tGFP* mice were induced with tamoxifen, and sections from the gastric cardia examined by immunofluorescence at 24 hours, 10 days, 20 days, 30 days, and 60 days after tamoxifen induction. Red: Non-recombined cells. Green: recombined cells. Blue: DAPI.

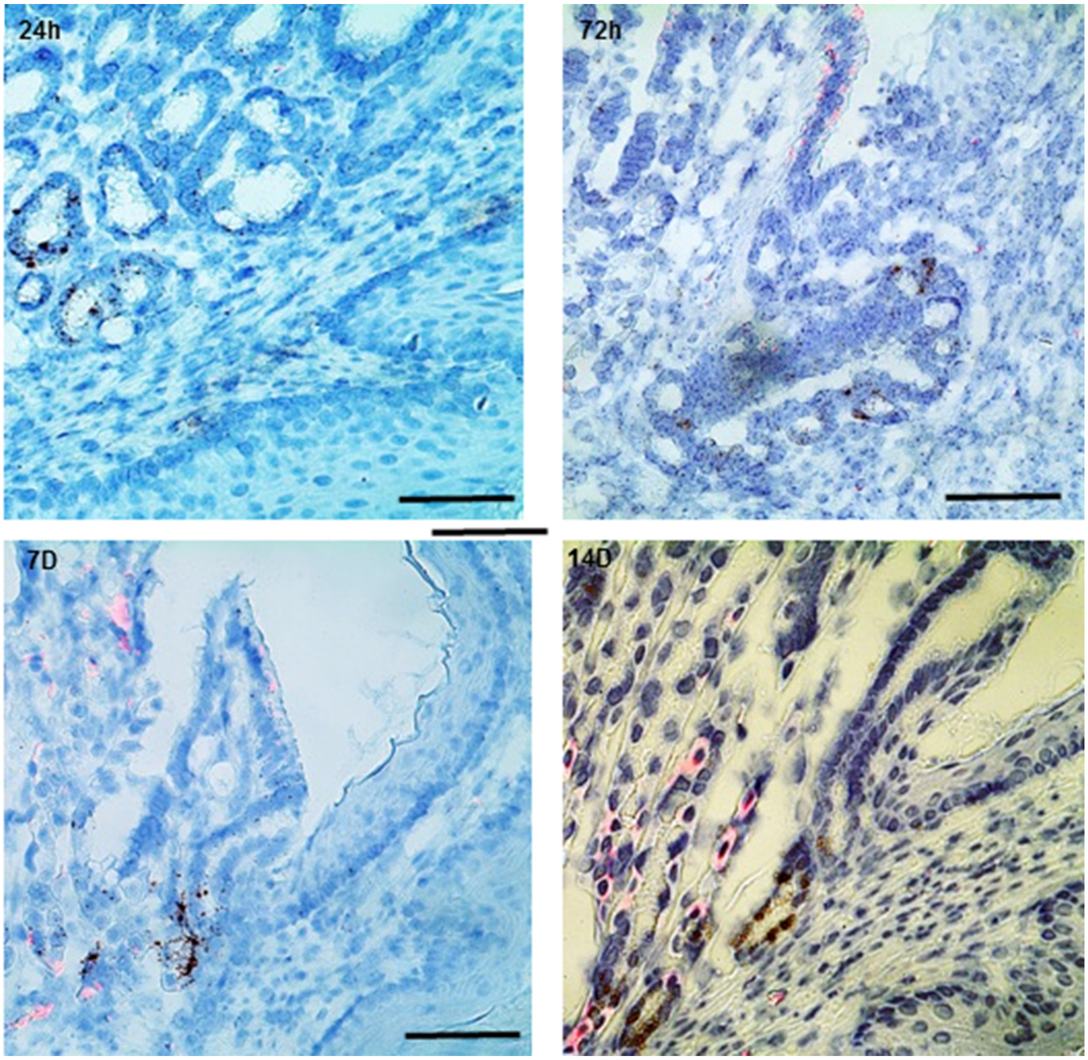

Supplementary Figure S4: CCK2R+ cardia cells give rise to Lgr5+ cells. RNAscope *in situ* hybridization for Lgr5+ cells (brown) with respect to lineage traced CCK2R+ cells (red) in *Lgr5-DTR-eGFP/CCK2R-CreERT/TdTom* mice at 24 h, 72 h, 7 d and 14 d time points.
